# A critical evaluation of the PTW 2D‐ARRAY seven29 and OCTAVIUS II phantom for IMRT and VMAT verification

**DOI:** 10.1120/jacmp.v14i6.4460

**Published:** 2013-11-04

**Authors:** Mohammad Hussein, Elizabeth J. Adams, Thomas J. Jordan, Catharine H. Clark, Andrew Nisbet

**Affiliations:** ^1^ Department of Medical Physics Royal Surrey County Hospital NHS Foundation Trust Guildford Surrey UK; ^2^ Department of Physics University of Surrey Guildford Surrey UK; ^3^ National Physical Laboratory Teddington UK

**Keywords:** IMRT, VMAT, QA, detector arrays

## Abstract

Quality assurance (QA) for intensity‐ and volumetric‐modulated radiotherapy (IMRT and VMAT) has evolved substantially. In recent years, various commercial 2D and 3D ionization chamber or diode detector arrays have become available, allowing for absolute verification with near real time results, allowing for streamlined QA. However, detector arrays are limited by their resolution, giving rise to concerns about their sensitivity to errors. Understanding the limitations of these devices is therefore critical. In this study, the sensitivity and resolution of the PTW 2D‐ARRAY seven29 and OCTAVIUS II phantom combination was comprehensively characterized for use in dynamic sliding window IMRT and RapidArc verification. Measurement comparisons were made between single acquisition and a multiple merged acquisition techniques to improve the effective resolution of the 2D‐ARRAY, as well as comparisons against GAFCHROMIC EBT2 film and electronic portal imaging dosimetry (EPID). The sensitivity and resolution of the 2D‐ARRAY was tested using two gantry angle 0° modulated test fields. Deliberate multileaf collimator (MLC) errors of 1, 2, and 5 mm and collimator rotation errors were inserted into IMRT and RapidArc plans for pelvis and head & neck sites, to test sensitivity to errors. The radiobiological impact of these errors was assessed to determine the gamma index passing criteria to be used with the 2D‐ARRAY to detect clinically relevant errors. For gamma index distributions, it was found that the 2D‐ARRAY in single acquisition mode was comparable to multiple acquisition modes, as well as film and EPID. It was found that the commonly used gamma index criteria of 3% dose difference or 3 mm distance to agreement may potentially mask clinically relevant errors. Gamma index criteria of 3%/2 mm with a passing threshold of 98%, or 2%/2 mm with a passing threshold of 95%, were found to be more sensitive. We suggest that the gamma index passing thresholds may be used for guidance, but also should be combined with a visual inspection of the gamma index distribution and calculation of the dose difference to assess whether there may be a clinical impact in failed regions.

PACS numbers: 87.55.Qr, 87.56.Fc

## I. INTRODUCTION

Intensity‐modulated radiotherapy, delivered using step‐and‐shoot or dynamic sliding window multileaf collimators (MLC), has facilitated complex treatments whereby the dose can be conformed to a target volume whilst minimizing the dose to surrounding normal tissue.^(1)^

Volumetric‐modulated arc therapy (VMAT), which combines dynamic MLC delivery along with gantry speed and dose rate modulation, has been a relatively new development.^(^ ^2^ ^,^ ^3^ ^)^ As with the treatment capabilities, quality assurance (QA) for IMRT and VMAT has evolved substantially to meet the increasing treatment complexity. In recent years, various commercial 2D and 3D ionization chamber or diode detector arrays have become available, allowing for verification of absolute dose with immediate results. Conventional methods, such as ionization chamber point dose measurements and film dosimetry, are gradually being replaced by detector arrays. These devices have allowed centers to streamline their QA and increase the number of patients treated with IMRT and VMAT. However, detector arrays are limited by their resolution, giving rise to concerns about their sensitivity to errors.

Commercial detector arrays available include the ${\text{Delta}}^{4}$ (ScandiDos AB, Uppsala, Sweden), ArcCHECK/MapCHECK (Sun Nuclear Corp., Melbourne, FL), MatriXX (IBA Dosimetry GmbH, Schwarzenbruck, Germany), and 2D‐ARRAY seven29 (PTW, Freiburg, Germany). Various studies have previously been performed to assess the sensitivity to IMRT and/or VMAT simulated errors for the ${\text{Delta}}^{4}$,^4^, ^5^, ^6^ ArcCHECK,^(^ ^5^ ^,^ ^7^ ^)^ and MapCHECK.^(^ ^8^ ^,^ ^9^ ^)^ For the OCTAVIUS II octagonal phantom and 2D‐ARRAY seven29 combination there is limited data available for composite plan verification for dynamic IMRT or Varian RapidArc (Varian Medical Systems, Palo Alto, CA). Masi et al.^(6)^ compared the 2D‐ARRAY seven29 with other dosimetry systems, including ${\text{Delta}}^{4}$ and MapCHECK, in the detectability of MLC errors in Elekta VMAT (Elekta, Stockholm, Sweden). Spezi et al.^(10)^ and Poppe et al.^(11)^ found that for step‐and‐shoot IMRT, 1 mm MLC errors could be detected for per‐beam planar verification using the 2D‐ARRAY.

The purpose of this study was to systematically characterize the sensitivity and resolution of the PTW 2D‐ARRAY seven29 and OCTAVIUS II phantom combination, using simulated errors, for optimal use in composite clinical dynamic IMRT and Varian RapidArc verification, including a comparison between a multiple 2D‐ARRAY acquisition technique to improve the effective resolution of the 2D‐ARRAY, and with the single acquisition technique. Comparisons were also performed against GAFCHROMIC EBT2 film (International Specialty Products, Wayne NJ).

## II. MATERIALS AND METHODS

### A. PTW 2D‐ARRAY seven29 and OCTAVIUS II phantom

The PTW 2D‐ARRAY seven29 consists of a matrix of 729 cubic vented ionization chambers with 0.5cm×0.5cm cross section, spaced 1 cm center‐to‐center, giving a total area of 27cm×27cm.^(12)^ The upper electrode layer sits below a 0.5 cm polymethyl methacrylate (PMMA) build‐up layer, whereas the lower electrode layer lies on top of a 0.2 cm thick electrode plate which itself is mounted on a 1 cm PMMA base plate. The OCTAVIUS phantom has an octagonal shape in its cross section, and is designed to allow composite rotational IMRT plan verification. The phantom is made of polystyrene which has a physical density of 1.04g/cm^3^. Its dimensions are 32 cm wide, 32 cm long, and 32 cm in height, and has a $30\times 30\;x\;2.2\;{\text{cm}}^{3}$ central cavity for the 2D‐ARRAY.^(13)^ Throughout this study, the OCTAVIUS II phantom was used in the coronal (horizontal) orientation.

**Figure 1:**
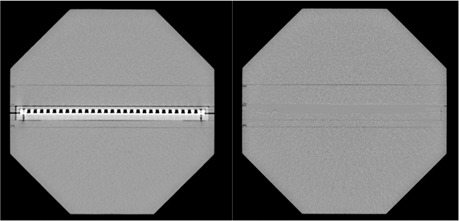
CT scan of the OCTAVIUS phantom with 2D‐ARRAY (left) *in situ* and homogeneous insert (right).

The OCTAVIUS phantom was CT scanned twice, once with the 2D‐ARRAY in situ and once with a homogeneous insert, for comparison (Fig. 1). For composite field measurements, the base of the OCTAVIUS contained a semicircular air gap to correct for the inherent under‐response of the 2D‐ARRAY when the radiation field is incident posteriorly, as described by Van Esch et al.^(13)^ For planning, the phantom was scanned with a solid base.

#### A.1 Basic commissioning tests

Basic commissioning tests of the 2D‐ARRAY were performed on a Varian Clinac iX (Varian Medical Systems). The Clinac incorporates the Millennium 120‐leaf MLCs, with the central 80 MLCs covering 20×20cm, each having a 0.5 cm width at the isocenter; the remaining MLCs have 1 cm width. The methodology used was in keeping with previously published reports and is, therefore, not discussed in detail.^(^ ^10^ ^,^ ^12^, ^13^, ^14^ ^)^ The effective point of measurement (EPOM) was determined following the methodology of Poppe et al.^(12)^ and Van Esch et al.^(13)^ Dose linearity was determined between 5–2500 cGy. Dose rate linearity was checked for dose rates ranging from 100–600 MU/min. Output versus field size were checked for 2×2 through to 25×25 cm field sizes. Profiles for both plain and wedged field sizes of 5×5cm,10×10cm, and 25×25cm were measured by the 2D‐ARRAY and compared with diode data measured in a Scanditronix Wellhöfer RFA water tank (IBA Dosimetry GmbH, Schuarzenbruck, Germany) at the same depth. For all the tests, except EPOM, the setup was such that the effective depth of the 2D‐ARRAY was at 5 cm. The beam energy used throughout was 6 MV (TPR20/10=0.670), focus‐to‐surface distance (FSD) was 100 cm, and 100 MU was used in all cases, except the dose linearity check. For the linearity measurements, a 10×10cm field size was used. Furthermore, the detectors within the 2D‐ARRAY have a relative calibration against the central detector and this was confirmed by setting an isocentric field with a 27×27cm field size to cover all the detectors within the 2D‐ARRAY and assessing uniformity by looking at a profile through each line of detectors, compared to a water tank profile.

#### A.2 Cross‐calibration procedure

The 2D‐ARRAY was calibrated using a cross‐calibration procedure. In this procedure a known dose was delivered and the response of the central detector was used to calculate a crosscalibration factor. This factor was applied to the entire matrix. For planar measurements, the 2D‐ARRAY was set up at an effective depth of 5 cm in solid water (Gammex Inc., Middleton, WI), and with 10 cm solid water backscatter. For composite measurements, the 2D‐ARRAY was setup within the OCTAVIUS phantom and the cross‐calibration procedure was performed in those conditions.

#### A.3 *Comparisons of using the OCTAVIUS scan with 2D‐ARRAY* in situ *vs. homogeneous scan*

Calculating on the scan of the OCTAVIUS with the 2D‐ARRAY *in situ* with an advanced calculation algorithm may result in perturbation of the predicted dose by the air‐filled ion chambers, which may add to uncertainties, particularly when using the gamma index analysis.^(15)^ Therefore, a dosimetric comparison was performed between using the OCTAVIUS scan with the 2D‐ARRAY *in situ* and a homogeneous insert.

Firstly, the directional response of the OCTAVIUS phantom was assessed by delivering a 10×10cm field in 15° gantry angle increments at 6 MV with the phantom set up isocentrically. The dose to the central detector was recorded. To avoid irradiating through the couch, the sectors comprising the first 180° were measured with the OCTAVIUS phantom in the normal setup, and the remaining sectors were measured by inverting the phantom. The expected dose at the central detector was calculated in the Varian Eclipse v8.9 treatment planning system (Varian Medical Systems) using the analytical anisotropic algorithm (AAA) v8.9 algorithm^(16)^ for both scans.

All clinical plan composite measurements, described below in the Materials & Methods section C.2, were evaluated using predicted doses calculated on both scans to compare the sensitivity of the gamma index analysis.

### B. Multiple acquisition modes in the 2D‐ARRAY

In the PTW VeriSoft software, it is possible to merge multiple measurement acquisitions, as proposed by Spezi et al.^(17)^ The sequence of measurements is as follows:

1. a measurement is performed at the central axis;
2. the 2D‐ARRAY is moved 0.5 cm inferior;
3. the 2D‐ARRAY is shifted 0.5 cm to the right; and
4. the 2D‐ARRAY is shifted 0.5 cm superior.

By performing the above sequence and merging the measurements, it was possible to effectively increase the total number of measurement points four‐fold from 729 to 2916, and improve the detector spacing from 1 cm to 0.5 cm center‐to‐center. For planar measurements, this can be easily achieved by automated couch movements. However, for composite measurements using the OCTAVIUS phantom, the 2D‐ARRAY must be shifted within the phantom and an insert is available to facilitate this. It may not be practical to perform this for every clinical plan verification. Therefore, the effect of different acquisition techniques was compared. All of the test fields and clinical plans described below were measured using the multiple acquisition technique. Comparisons were then performed between:

- single acquisition
- merging in the lateral direction only (by performing two acquisitions)
- merging in the longitudinal direction only
- full merge after four acquisitions

**Figure 2:**
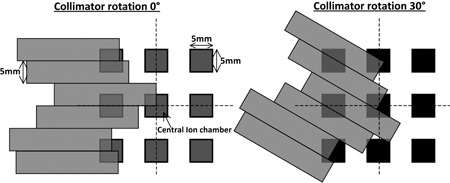
Schematic of the overlap between the 5 mm Varian Millenium MLCs and ion chambers within the 2D‐ARRAY for a collimator rotation of 0° (as typically used for static gantry IMRT) and 30° (as typically used for RapidArc). The dashed line indicates the central axis.

The gap between each ion chamber in the 2D‐ARRAY is 5 mm wide. as can be seen in the schematic in Fig. 2. Suppose that only a single 5 mm MLC leaf was being sampled. In this case, three possibilities may occur for a collimator rotation of 0°: i) direct overlap between the leaf and a line of detectors, ii) partial overlap with a line of detectors, or iii) complete miss if the leaf aligns with the gap between the lines of detectors.

In normal situations, whereby the setup is such that the field's crosshairs align with the marks on the 2D‐ARRAY and OCTAVIUS phantom, the central axis will intersect the central detector. In this case, scenario ii will occur (illustrated in Fig. 2) for static gantry IMRT, where the collimator is typically set to 0°, and for RapidArc, where the collimator angle may be typically set to 30°. However, scenario i and iii above would occur if a superior‐inferior movement of 0.5 cm is performed. In this case, every other MLC leaf will directly overlap with a row of detectors for a collimator angle of 0°, and the remainder will be missed. This effect is minimized where there is a collimator rotation. Therefore in order to test the limits of the 2D‐ARRAY, comparisons were also performed using acquisition #2 in the measurement sequence described above.

### C. Deliberate errors

The resolution and sensitivity of the 2D‐ARRAY was tested by a number of methods. All plans described in the following subsections were created using Eclipse (Varian Medical Systems), and calculations were performed using the AAA with a 0.25 cm grid spacing.

Measurements were performed on the same Varian Clinac iX over two sessions. Plans were generated to make optimal use of the 0.5 cm MLCs. The 2D‐ARRAY was cross‐calibrated in the morning and afternoon of each session to account for any output fluctuation. In all cases, the normal plan (i.e., with no errors) was measured for baseline. Measurements were also performed using GAFCHROMIC EBT2 film in the OCTAVIUS phantom in the same plane as the 2D‐ARRAY, using the film insert provided with the phantom. In the case of the film measurements, the solid OCTAVIUS base was used and plans were calculated on a homogeneous scan. GAFCHROMIC films were processed and analyzed 24 hours after exposure. Films were scanned using the Epson Espression 10000 XL flatbed colour scanner (US Epson, Long Beach, CA) at a resolution of 75 dpi, using the red channel.^(18)^

In all cases, the gamma index (y) method of evaluation was used with a 20% threshold.^(15)^ Various criteria for γ were analyzed, including the commonly used 3% dose difference and 3 mm distance‐to‐agreement (DTA) criteria. For the 2D‐ARRAY, analysis was performed using the PTW VeriSoft software version 4.1. For film, analysis was performed using the IBA OmniPro‐I'mRT software version 1.7. Both the film and plan data were normalized to 100% at a point in a high‐dose low‐gradient region, to perform a relative comparison. This procedure is commonly used for film analysis due to the known difficulty in performing an absolute dose calibration for film.^(19)^ In order to maintain a consistent comparison, the 2D‐ARRAY data was also rescaled in the same way as the film. In both cases, the normalization point for the gamma analysis was kept consistent for any particular set of measurement; for example, in the prostate IMRT plan with different errors introduced, the normalization value was always kept the same to avoid bias.

#### C.1 Gantry angle 0° test fields

As a starting point, it was necessary to understand the limitations of the 2D‐ARRAY in its basic IMRT measuring mode — that is setting the gantry to 0° and delivering a modulated field such that the 2D‐ARRAY is orthogonal to the beam. The aim was to investigate the two following questions:

- How does the detector spacing of the 2D‐ARRAY affect the measurement and visualization of a highly modulated field?
- How does the detector spacing affect the sensitivity of the gamma index in a modulated field?

Therefore, two individual planar test fields were designed specifically with the aim of addressing these questions. The test fields described below were also measured using the Varian aS1000 Electronic Portal Imaging Device (EPID) and GAFCHROMIC film.

**Figure 3:**
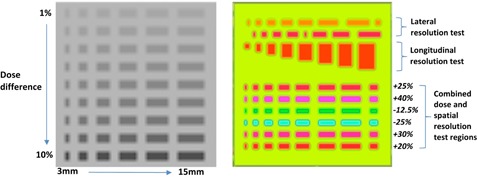
Single gantry test fields for sensitivity (left) and resolution (right) assessment. In the sensitivity test, the regions vary in width from left to right between 3 mm and 15 mm, and the dose difference relative to the background varies from top to bottom by 1 % to 10%. In the resolution test, the values in the lower half represent difference in percent dose between the regions and the background (lime green) area. In both tests, each row represents a single MLC leaf.

The first test field was designed to test the sensitivity of the gamma index analysis calculated in the 2D‐ARRAY using a modulated field with regions ranging from subtle to significant. This test will be referred to as the “sensitivity test”. The test had 54 regions of varying width and dose difference introduced into an open 15×15cm field using the fluence dose painting tool in Eclipse, as shown in Fig. 3 (left). The minimum spot size that the fluence painting tool allowed was 3 mm width and 5 mm height. As such, the columns in the test varied between one to six adjacent fluence spots (i.e., the width varied from 3 mm in the first columns up to 15 mm in the last column). Each row had a height of 5 mm and corresponded to a MLC leaf, and the gap between each region was 5 mm. The difference in dose between each row and the high‐dose background ranged between 1% and 10%. The measurement of the field was compared to the open field predicted dose to determine the minimum detectable error by means of the gamma index analysis. Parameters for the analysis were varied from 1%‐10% dose difference, and 1–3 mm DTA.

In the second test, a highly modulated field was created by dose painting varying dose and spatial information into an initially uniform field, as shown in Fig. 3 (right). This field is more complex than a field encountered clinically and tests the limits of the 2D‐ARRAY The first two lines in the field were offset from each other by 5 mm. This meant that due to the design of the 2D‐ARRAY, the resolution in the lateral direction could be tested. The third line increased in size in the longitudinal direction and, therefore, the resolution could be tested in this direction. The remaining six lines were used as a combined spatial and dose resolution test. This test will be referred to as the “resolution test”. The field was delivered to the 2D‐ARRAY to test how well it performs in distinguishing the regions.

#### C.2 Clinical IMRT and RapidArc plan errors

To assess the sensitivity and resolution of the 2D‐ARRAY in composite clinical plan verification using the OCTAVIUS phantom, a range of errors were simulated. Firstly, deliberate single MLC leaf positional errors of 1 mm, 2 mm, and 5 mm were introduced, consistently throughout the dynamic leaf motion, into pelvic and head & neck IMRT and RapidArc clinical plans to simulate subtle MLC mechanical errors. This was achieved by editing each control point within the RT Plan DICOM files. Measurements were evaluated against the TPS calculated dose distribution. Plans included five‐field prostate IMRT, five‐field head & neck IMRT, single arc prostate RapidArc, and two‐Arc head & neck RapidArc. All plan files were reloaded into the Eclipse TPS and calculated to ensure the fields were deliverable. The test fields were delivered using the same monitor units (MUs) as the original plan and compared against the original unedited plan.

Deliberate collimator rotation errors of 1°, 2° and 5° were also introduced into a two‐arc prostate and pelvic nodes RapidArc plan. A further test was created using the fluence editing tool in Eclipse to manually paint hot and cold dose spots of varying dimensions into a field in a prostate and pelvic node IMRT plan. In this case a recalculation of the leaf sequence was required; however, the intention with this test was to introduce significant errors to test the sensitivity and the resolution of the 2D‐ARRAY.

All clinical error plans were also calculated on the OCTAVIUS scan with homogeneous insert. This meant it was possible to simulate the expected gamma index pass rate in ‘ideal’ conditions (i.e., no output fluctuation and no inherent mechanical effects on the other unperturbed MLC leaves). This was done by exporting the normal plan predicted dose plane and the dose plane for each error plan. The expected gamma index was calculated in both VeriSoft and OmniPro to check how well they agreed. The expected passing rate for each plan was taken as the average of the VeriSoft and OmniPro calculation.

#### C.3 Effect of normalization point

The effect of choosing a point for the gamma index evaluation was investigated to assess whether there would be an influence on results. The analysis described above was repeated by deliberately choosing a dose point in a region where there was an MLC error, and by choosing the dose based on a mean value over the high‐dose region. In order to facilitate the latter, a custom spreadsheet was generated in Microsoft Excel 2007 (Redmond, WA). The spreadsheet was created such that it was possible to import the 2D‐ARRAY measurement and predicted dose. The local percent dose difference was calculated on a per detector basis, by comparing the measurement against the corresponding predicted dose. Customizable thresholds were also written into the spreadsheet such that the user may choose a lower and upper threshold for any value between 0% and 100%. The maximum dose point was taken as 100%. Assuming that the ICRU Report 83^(20)^ conditions were met for the high‐dose region, the coverage would range between 95% and 107% of the prescribed dose (i.e., a range of 12%). Allowing for changes in the homogeneity when the plan was transferred to the OCTAVIUS phantom, a lower threshold of 85% was used to ensure complete sampling of the primary PTV region. This spreadsheet was generated because it was found that the commercial systems (VeriSoft and OmniPro) limited the lower threshold to a maximum of 30%, whether for dose difference or gamma analysis. It was then possible to acquire various statistics such as the mean of all the dose differences and standard deviation. The spreadsheet was also written in a way that a comparison may be performed between any predicted dose plane versus another.

### D. Dosimetric and radiobiological evaluation of clinical plan errors

The dosimetric impact of the subtle MLC errors was assessed using the spreadsheet described above. The predicted dose due to a MLC error was exported to compare against the unperturbed predicted dose. The expected local dose difference caused by the deliberate MLC errors was calculated and compared to that found by the 2D‐ARRAY. Additionally, the mean dose difference over a high‐dose region was also calculated.

In addition to the dosimetric impact, it was also possible to determine whether there is a theoretical radiobiological effect due to the errors introduced into the clinical plans, as described by Carver et al.^(4)^ Tumor control probability (TCP) calculations and normal tissue complication probabilities were performed in BIOPLAN.^(21)^ TCP was calculated using the mechanistic Poisson‐based TCP model.^(22)^ The following input parameters for the TCP models for prostate tumors were used: radiosensitivity parameter $\alpha =0.29\;{\text{Gy}}^{-1}$, interpatient variation in radiosensitivity parameter $\alpha =0.07\;{\text{Gy}}^{-1}$, clonogenic cell density $\rho_{c}=107\;{\text{cm}}^{-3}$, as well as an α/β ratio of 10 Gy.^(23)^ For squamous cell carcinoma, parameters were chosen as $\alpha /\beta =10\;\text{Gy},\;\alpha =0.305\;{\text{Gy}}^{-1},\;\alpha =0.07\;{\text{Gy}}^{-1},\;\rho_{c}=107\;{\text{cm}}^{-3}$.^(24)^

In the prostate cases, NTCP calculations were performed for the rectum using the Lyman‐Kutcher‐Bauman model,^25^, ^26^, ^27^ generalized uniform dose concept,^(28)^ and Quantative Analysis of Normal Tissue Effects in the Clinic recommended best parameter estimates of α/β=3Gy, volume effects parameter (n)=0.09, slope parameter (m)=0.13, and the dose for 50% complication probability (TD50)=76.9Gy. For the bladder, there is limited NTCP parameter data due to difficulties in fitting parameters to genitourinary toxicity.^(29)^ The general consensus is to use the parameters of n=0.5,m=0.11,andTD50=80Gy, in conjunction with α/β=3Gy.^(30)^ In the head & neck plans, NTCP values were calculated for spinal cord and parotids. For spinal cord, parameters for myelopathy were taken as α/β=3Gy,n=0.05,m=0.175,TD50=66.5Gy.^(30)^ For xerostomia, α/β=3Gy,n=0.7,m=0.18,TD50=46Gy.^(30)^

### E. Data and statistical analysis

To perform a quantitative analysis between the different permutations described above, a range of gamma index^(15)^ passing criteria was recorded, including the commonly used 3%/3 mm. For each passing criteria, the percentage of detectors with γ<1 was recorded. We propose an analysis technique, whereby cumulative histograms of the percentage of planes measured against the percentage of detectors/pixels passing with γ<1 are plotted. This makes it possible to compare the sensitivity of different passing criteria and different measurement technique qualitatively.

In addition to the above analysis method, agreement between passing rates in the different measurement permutations and the 2D‐ARRAY measurement in single acquisition mode was statistically assessed using the concordance correlation coefficient, $p_{c}$.^(31)^ In the case of poor agreement, the statistical significance of any difference was assessed using the Wilcoxon signed‐rank test with p<0.05 as the threshold for significance.

## III. RESULTS

### A. Basic commissioning

Dose linearity between 5–2500 cGy had a Pearson correlation coefficient, r, of 1.0. Dose rate linearity was found to be within ±0.2% between 100–600 MU/minute. Profiles of field sizes ranging from 4×4 to 25×25cm and wedged profiles measured with the 2D‐ARRAY agreed within measurement uncertainty with profiles measured using a diode in a water tank (the concordance correlation coefficient, $\rho_{c}$, was >0.995 for all). Basic commissioning data agreed well with previously reported data.^(^ ^10^ ^,^ ^12^, ^13^, ^14^ ^)^ The EPOM was measured to be 0.75 cm from the surface, in agreement with the operational manual and published data.^(13)^

#### A.1 *Directional response in the OCTAVIUS scan with 2D‐ARRAY* in situ *vs. homogeneous scan*

**Figure 4:**
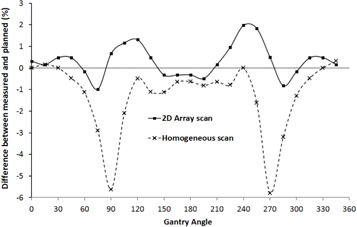
Angular response of the 2D‐ARRAY; comparison using predicted doses in the 2D‐ARRAY scan and in the homogeneous scan.

The result of the directional response evaluation can be seen in Fig. 4. The graph shows the difference between the measured and expected dose in the central detector within the 2D‐ARRAY as a function of gantry angle. The difference between the total dose given to the central detector and expected was 0.3% when the 2D‐ARRAY was scanned in the OCTAVIUS phantom and was −1.3% when a homogeneous insert was used. It can be seen that using a homogeneous scan results in a significant underresponse when the beam incidence is lateral or entering the ARRAY through an oblique direction. This is due to the lack of modeling of the inhomogeneities caused by the vented ion chambers within the 2D‐ARRAY. When the scan of the 2D‐ARRAY was used, this improved the response. It is worth noting that this comparison is reported for the AAA algorithm. Calculating on the 2D‐ARRAY scan using the pencil beam convolution algorithm with heterogeneity correction yielded, as expected, a similar result to that seen with the homogeneous insert calculated using AAA.

### B. Gantry angle 0° test fields

**Figure 5:**
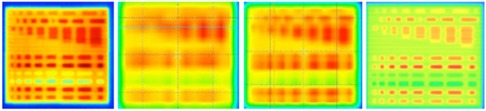
(left to right) GAFCHROMIC film of resolution test, 2D‐ARRAY measurement (single acquisition), 2D‐ARRAY measurement (fully merged), EPID measurement.

Measurements of the resolution test field for the 2D‐ARRAY in single acquisition and full merge mode, GAFCHROMIC film, and EPID are shown in Fig. 5. The 2D‐ARRAY was able to distinguish dose differences, but there was a smoothing effect in the single acquisition which was less apparent when the effective resolution was improved to 5 mm.

**Figure 6:**
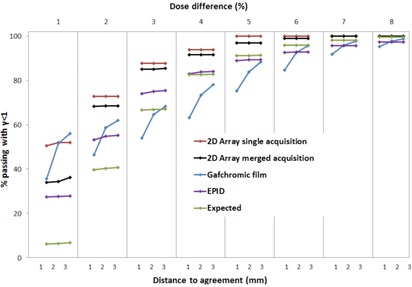
Gamma index passing rates for the sensitivity test field for different measurement permutations. Points have been linked to provide a visual guide.

Passing rates for the sensitivity test using various gamma index criteria are shown in Fig. 6. Data have been plotted for the 2D‐ARRAY in single and full merge acquisition modes, film, EPID, and expected passing rate. It can be seen that the DTA criteria had a minimal impact in this test field for all the permutations, except for the GAFCHROMIC film. The single acquisition 2D‐ARRAY data can be seen to be the least sensitive when compared to the expected passing rate; however, this improved when a full merge was performed. As the dose difference criterion was increased, the different systems began to converge. The EPID measurement was found to be the most sensitive for planar field measurements.

### C. Dosimetric and radiobiological impact of the deliberate composite clinical plan errors

**Figure 7:**
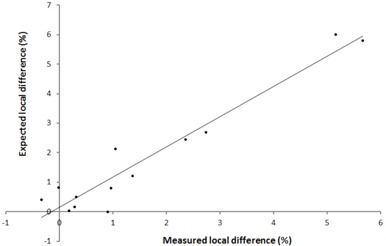
Expected vs. measured local dose difference due to the MLC errors.

The ability of the 2D‐ARRAY to detect local dose differences caused by the MLC errors is shown in Fig. 7. There was a statistically good agreement between the dose difference detected by the 2D‐ARRAY and the expected difference $\left(\rho_{c}=0.96\right)$. A 1 mm MLC error caused up to a 1% local dose difference whereas, for a 2 mm error, this was between 1% and 3% and for a 5 mm MLC error, local dose difference of between 3% and 6% was observed.

In the prostate IMRT plan, a 5 mm MLC error resulted in a 0.4% rectal NTCP increase, whereas in the RapidArc plan, this was 1.2%. For a 2 mm error, the increase in the rectal NTCP for IMRT and RapidArc plans was 0.3% and 0.9%, respectively. In the prostate and nodes plan with collimator rotation errors, a 1° and 2° error resulted in an increased rectal NTCP of 3.0% and 3.2%, respectively. In all prostate plans, bladder NTCPs were found to be 0%, although this may not be clinically relevant and is due to the difficulty of fitting parameters to genitourinary toxicity.^(29)^ In the head & neck plans, NTCP values for spinal cord did not increase and were 0.2% for all IMRT plans and 0.1% for all RapidArc plans; these values are in keeping with published data on the incidence of myelopathy at the 45 Gy level.^(32)^ Similarly for the parotids, the maximum increase was limited to 0.2%. As expected, TCP values increased in all plans due to the increase in local dose from the MLC errors. This was as high as an increase of 3% for a 5 mm MLC error and a collimator rotation error of 2°.

### D. Composite verification of clinical plans

All the unperturbed plans had a γ<1 passing rate of 100% using 3%/3 mm. When using 2%/2 mm, the passing rate for all the plans was greater than 97%. The 5 mm systematic MLC errors were detected using 3%/3 mm in the 2D‐ARRAY in the IMRT plans and in the prostate single‐arc RapidArc plans. However, the 2 mm systematic errors were difficult to detect using 3%/3 mm; the γ in the region where the errors occurred was increased in comparison to the surrounding area, but was still <1, and hence would not be detected as a fault. The error was detectable at 2%/2 mm. For the head & neck two‐arc RapidArc plan, none of the errors were visible in the measurement and were also found to have a low impact in the expected gamma index maps. This is due to the plan having opposing collimator rotations on each arc to minimize the tongue‐and‐groove effect and the errors may have been largely cancelled out. For the prostate and pelvic nodes RapidArc plan with collimator rotation errors, 3%/3 mm gave a passing rate of >99% for deliberate 1° and 2° errors, and reduced to 92% in the presence of a 5° error. The 1° error would have still passed at 2%/2 mm with a passing rate of 99.3%. The 2° error, however, resulted in a passing rate of 94.1% and would have failed if a 95% threshold was used. At 2%/2 mm, the passing rate for the 5° error plan was 74.6%. In the cases where the deliberate errors were detectable using 2%/2 mm, a passing criteria of 3%/2 mm would have passed if a passing threshold of 95% was used; however, had a passing threshold of 98% been used, then these measurements would have failed. Table 1 gives a summary of the average and minimum percentage of detectors/pixels passing with γ<1 in all the plans, for 3%/3 mm, 3%/2 mm, and 2%/2 mm passing criteria for the different acquisition permutations.

The analysis of the effect of choosing a normalization point found that there was no significant difference between choosing a point in an unperturbed region, a point in an error region, or mean dose within the 85% isodose at 3%/3 mm or 3%/2 mm. At 2%/2 mm, there was a reduction in the passing rate in the analysis based on mean dose by 0.5% compared to the other two normalization techniques. This reduction was small but statistically significant (p=0.001).

**Table 1:** Summary of mean and minimum gamma index passing criteria for all various measurement permutations. A lower number indicates greater sensitivity to error detection. The concordance correlation coefficient, $\rho_{c}$, is also given, assessing agreement with single 2D‐ARRAY acquisition

		*Percent Detectors/Pixels Passing with* γ<1 *and* $\rho_{c}$								
		*3%/3 mm*			*3%/2 mm*			*2%/2 mm*		
*Device*	*Acquisition*	*Mean*	*Min*	$\rho_{c}$	*Mean*	*Min*	$\rho_{c}$	*Mean*	*Min*	$\text{Pc}\rho_{c}$
2D‐ARRAY	Single	98.9	92.0	−	97.9	82.6	−	94.7	74.6	−
	Merged lateral	98.8	91.3	0.984	97.8	82.0	0.979	95.0	74.0	0.957
	Merged longitudinal	98.6	88.8	0.948	97.6	80.9	0.967	94.6	72.3	0.966
	Merged full	98.6	89.5	0.922	97.5	80.9	0.941	94.8	72.1	0.912
	Shift 5 mm longitudinal^a^	98.5	85.7	0.875	97.8	79.2	0.959	95.1	69.9	0.910
	Homogeneous scan	99.0	91.8	0.851	98.3	86.6	0.795	96.1	74.6	0.703
GAFCHROMIC film		98.5	95.5	0.204	95.4	87.8	0.060	92.1	81.3	0.062

a Acquisition number 2 as described in the Material & Methods section B.

### E. Comparison between expected gamma index passing rates in VeriSoft and OmniPro

There was good statistical agreement between the expected gamma index passing rates calculated in VeriSoft and OmniPro as indicated by the concordance correlation coefficient ($p_{c}>0.90$ for all passing criteria). The average difference between the passing rates calculated by VeriSoft and OmniPro was 0.5% and 1.1% for 3%/3 mm and 2%/2 mm criteria, respectively. The difference was found to be statistically not significant (p>0.20 for all). It was therefore reasonable to use the average passing rate for the expected gamma index calculated by both software for each clinical plan to compare against that measured by 2D‐ARRAY and GAFCHROMIC film.

### F. Comparison Between Evaluations using CT Scan with 2D‐Array *in situ* vs. Homogeneous Insert

**Figure 8:**
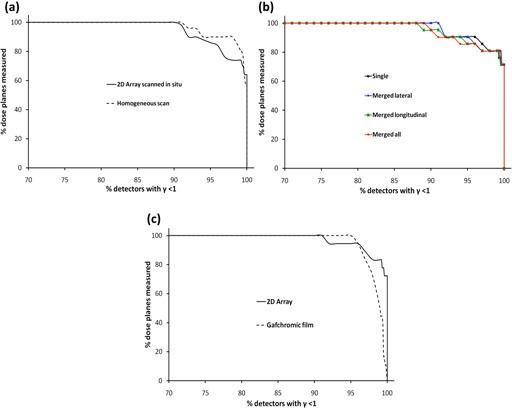
Cumulative histogram of gamma index analysis using a passing criteria of 3%/3 mm for: (a) comparison of using 2D‐ARRAY *in situ* scan vs. homogeneous scan, (b) comparison of different acquisition techniques, and (c) comparison of 2D‐ARRAY vs. GAFCHROMIC film measurements.

As shown in Fig. 8(a) and Table 1, there was a small difference between passing rates using a criteria of 3%/3 mm. However, at 2%/2 mm, using the scan with the 2D‐ARRAY *in situ* appeared to be more sensitive to errors than comparing against the predicted dose calculated on the homogeneous scan; for criteria of 2%/2 mm, the average passing rate was 95.7% compared to 88.5% in the 2D‐ARRAY scan.

### G. Single vs. multiple 2D‐ARRAY acquisition modes

**Figure 9:**
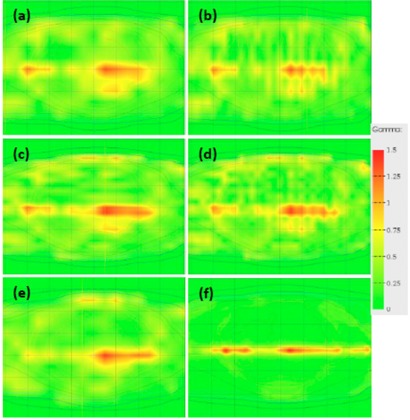
Gamma maps using 3%/3 mm criteria showing the effect of multiple acquisition modes for prostate IMRT plan with 5 mm MLC error: (a) single acquisition, passing rate 96.8%; (b) two merged acquisitions with array shifted lateral for second acquisition, 97.0%; (c) two merged acquisitions with array shifted longitudinal for second acquisition, 96.2%; (d) four merged acquisitions to give effective 5 mm resolution, 96.8%; (e) acquisition with 5 mm shift in the longitudinal direction, 96.1%; and (f) predicted gamma index distribution, 99.5%.

There was no significant difference in the gamma index passing rate, at either 3%/3 mm or 2%/2 mm, between performing single or multiple acquisitions, as shown in the cumulative histogram in Table 1 and in Fig. 8(b) for (3%/3 mm). Figure 9 shows the gamma index maps and passing rates for the prostate IMRT plan with a 5 mm MLC error for single versus multiple acquisitions. It can be seen that performing a merged lateral acquisition is visually comparable to a single acquisition, whereas slightly improved resolution is achieved by either merging longitudinally or performing a full merge of four acquisitions. It can also be seen in Fig. 9 and Table 1 that the single acquisition was comparable to the acquisition shifted 5 mm on the longitudinal axis, demonstrating no significant reduction in sensitivity to errors. Overall the acquisition shifted 5 mm longitudinal was found to be the most sensitive acquisition position, based on the gamma index passing rates.

### H. 2D‐ARRAY vs. GAFCHROMIC film

**Figure 10:**
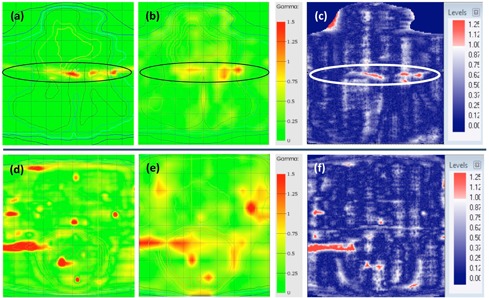
Comparison between gamma index distribution at a passing criteria of 3%/3 mm for the head & neck IMRT plan with 5 mm MLC error: (a) predicted, (b) 2D‐ARRAY, (c) GAFCHROMIC film; and for the prostate & nodes plan with randomly distributed errors for: (d) predicted, (e) 2D‐ARRAY, (f) film.

Figure 10 shows a comparison between the gamma index distribution (using 3%/3 mm) in the 2D‐ARRAY, GAFCHROMIC film, and expected gamma index distribution for the head & neck IMRT plan with a 5 mm MLC error and prostate and nodes with randomly distributed errors. Regions of failure were comparable between the 2D‐ARRAY and GAFCHROMIC film, with the 2D‐ARRAY exhibiting the blurred effect due to its resolution. Neither system picked up all the errors in the prostate and nodes plan with random errors.

Average and minimum gamma index passing rates using criteria of 3%/3 mm were comparable for the 2D‐ARRAY and film, as shown in Table 1 and the cumulative histogram in Fig. 8(c). At 2%/2 mm the 2D‐ARRAY resulted in a higher overall passing rate. For a passing rate of 85% or below, the 2D‐ARRAY and GAFCHROMIC film were comparable at 2%/2 mm. For passing criteria of 3%/3 mm, all film planes achieved 95% passing rate or above; for the 2D‐ARRAY, this was found to be 90.5% of measured planes. At 2%/2 mm 33.3% of film planes achieved a passing rate of 95% or above, whereas for the 2D‐ARRAY it was 66.7%. Statistically, there was a poor agreement between 2D‐ARRAY and film as given by $\rho_{c}$ for each passing criteria. The difference between 2D‐ARRAY and GAFCHROMIC film was statistically significant for passing criteria 3%/3mmor3%/2mm(p=0.048and0.001,respectively), however it was not significant for 2%/2mm(p=0.11). When compared against the expected gamma passing rate, the 2D‐ARRAY result had a statistically more significant agreement $\left(\rho_{c}=0.91\;\text{for}\;3\%/3\;\text{mm},\;\text{and}\;0.79\;\text{for}\;2\%/2\;\text{mm}\right)$ than GAFCHROMIC film $\left(\rho_{c}=0.35\;\text{for}\;3\%/3\;\text{mm},\;\text{and}\;0.22\;\text{for}\;2\%/2\;\text{mm}\right)$.

## IV. DISCUSSION

For planar measurements of IMRT fields, the 2D‐ARRAY in single acquisition mode performed the worst in measuring the sensitivity and resolution test fields. Spatial resolution was significantly affected; however, dose resolution was less affected. This was due to the sparse resolution of 1 cm. Improvements were found when a full merge acquisition was performed. In measuring individual IMRT fields with the 2D‐ARRAY orthogonal to the beam, the resolution may be more influenced by the modulated nature of the fields. This may have less significance in a prostate plan than in a head & neck cancer case. It appeared that the GAFCHROMIC film measurement, although very good spatially, was giving false negative results in the sensitivity test. This is due to intrinsic film heterogeneity causing minor artifacts combined with processing uncertainty, which were enough to disrupt the gamma index analysis passing rate, and are some of the known limitations of film dosimetry.^(^ ^18^ ^,^ ^33^ ^)^ The EPID was found to be the most effective of the different devices. In this regard, if using a 2D‐ARRAY for planar field measurement, it would be advisable to consider performing a full merge acquisition when measuring very complex planar fields.

In composite plan verification, the 2D‐ARRAY demonstrated good sensitivity to subtle MLC errors. There was a reasonable comparison between the gamma index distributions generated by the 2D‐ARRAY and GAFCHROMIC film. At 3%/3 mm, passing rates were similar between the two systems. The 2D‐ARRAY did exhibit a higher passing rate at 2%/2 mm compared to film. However, it was also interesting to see that the passing rates from the 2D‐ARRAY agreed better with the expected passing rates than GAFCHROMIC film, consistent with the static gantry planar test fields. The effect of picking a normalization point was found to be minimal, but there was a statistically significant small difference between normalizing based on a mean dose and a point in the measured distribution. Performing a normalization based on a mean dose would provide more consistency.

Performing a merged lateral acquisition was visually comparable to a single acquisition, whereas slightly improved resolution was achieved by either merging longitudinally or performing a full merge of four acquisitions. This is because, in the case of a lateral shift, resolution is only gained along the MLC leaf path, whereas merging in the longitudinal direction perpendicular to the MLCs allowed more sampling of the leaf bank. The single acquisition was also comparable to the acquisition shifted 5 mm on the longitudinal axis, demonstrating no significant reduction in sensitivity to errors. The lack of difference between the different acquisition modes can be explained by the fact that on Varian linear accelerators, MLCs are arranged either side of the central axis. However, the 2D‐ARRAY is set up such that the central detector is aligned directly with the central axis. Therefore each chamber is always sampling two 5 mm MLC leaves simultaneously. A 5 mm offset in the longitudinal direction would result in every other MLC potentially being missed. It would, therefore, be recommended that if a longitudinal shift is required (e.g., for a long IMRT field where it is necessary to avoid irradiating the electronics), that the shift be made in whole centimeters. It also appears that calculating the expected dose on a homogeneous scan may be less sensitive to errors than calculating on a scan with the 2D‐ARRAY *in situ*. This is due to underestimation of the dose from the lateral and oblique directions when using the homogeneous scan.

Various studies have been performed on the impact of errors in different detector systems.^(^ ^4^ ^,^ ^5^ ^,^ ^7^ ^,^ ^34^ ^,^ ^35^ ^)^ At the time of writing, this was the first study to perform such a comprehensive evaluation of the PTW 2D‐ARRAY seven29 and OCTAVIUS II phantom combination, particularly for dynamic IMRT and RapidArc. These errors were designed to test the sensitivity and resolution of the 2D‐ARRAY. However, an interesting question to pose is: How likely are these errors in clinical practice? It is possible that an MLC motor becomes less efficient due to wear and tear, leading to a leaf travelling slower than expected and therefore lag behind the other leaves, in a way that would be similar to the errors simulated in this study. However, the tolerance on the MLC control software (usually about 2 mm) means that there are generally two possible scenarios: if possible, all the other leaves are slowed down and the dose rate decreased to compensate for the slower leaf, or an interlock would be activated. Software errors may also possibly lead to a mistranslation of the MLC positions. This study has demonstrated that in the event of MLC errors occurring, that the 2D‐ARRAY seven29 combined with the OCTAVIUS II phantom was a sensitive and reliable method of absolute IMRT or RapidArc verification.

The suitability of the gamma index in detecting errors that may be clinically significant has been previously criticized.^(36)^ Alternatives have been suggested.^36^, ^37^, ^38^, ^39^ It should be noted that the gamma index has been widely accepted and is implemented into most analysis software. It provides the means for an efficient analysis, particularly important within a busy clinical environment. It is a useful parameter when one performs an audit of IMRT QA over the past months or years in order to explore trends and attempt to improve or streamline QA. It is equally important to understand the limitations of the index combined with the equipment in use. There is a general trend to use 3%/3 mm with a 95% passing threshold. In this study, it was found that in terms of passing rate, the criteria of 3%/3 mm masked errors caused by deliberate collimator rotation errors of 1° and 2°, as well as 2 mm MLC errors. The collimator errors introduced in the prostate and nodes RapidArc plan caused the rectal NTCP to increase by about 3%, which may be clinically significant. The 2 mm errors increased the rectal NTCP up to 0.9% in the prostate plans. These errors were detectable using passing criteria of 2%/2 mm with a 95% threshold or using passing criteria of 3%/2 mm with a 98% passing threshold. For this system, these may be the recommend criteria to be used in order to detect errors that may cause a clinically significant increase in NTCP. The errors introduced all increased local dose difference and, therefore, the TCP was increased. One limitation of this study would be that none of the errors resulted in a reduction of TCP. All the MLC errors were designed to increase the leaf gap. We did not create errors with closed leaf gaps as there was a risk of causing MLC collisions. Errors with narrower leaf gaps would have been expected to cause dose reductions. It was shown in Fig. 7 that the 2D‐ARRAY was able to detect the dose differences caused by the MLC errors, and the strong linear relationship between the expected dose difference and the measured difference suggests that dose reductions may have been detected equally. We suggest that the gamma index passing thresholds be used for guidance, but also be combined with a visual inspection of the gamma index distribution and calculation of the dose difference to assess whether there may be a clinical impact in failed regions.

## IV. CONCLUSIONS

Tests have been employed to characterize the sensitivity and resolution of the PTW 2D‐ARRAY seven29 and OCTAVIUS II phantom combination. The 2D‐ARRAY in single acquisition mode was comparable to multiple acquisition modes and GAFCHROMIC film for composite IMRT and RapidArc plan verification. A gamma index criterion of 3%/3 mm may potentially mask clinically relevant errors. A criterion of 3%/2 mm with a passing threshold of 98% or 2%/2 mm with a passing threshold of 95% was found to be more sensitive in conjunction with an evaluation of the gamma index distribution. These tests have resulted in an understanding of the 2D‐ARRAY's limitations and increased confidence in its use for clinical IMRT and RapidArc verification.

## ACKNOWLEDGMENTS

The authors would like to thank Yatman Tsang (Mount Vernon Hospital, UK) for assistance with the processing of the GAFCHROMIC EBT2 film.
